# Spirulina ameliorates arsenic induced reproductive toxicity in male rats

**DOI:** 10.1590/1984-3143-AR2021-0035

**Published:** 2021-11-19

**Authors:** Abul Khair, Mohammed Abdul Awal, Mohammed Nazmul Hoque, Anup Kumar Talukder, Ziban Chandra Das, Damanna Ramkishan Rao, Mohammed Shamsuddin

**Affiliations:** 1 Quality Control Laboratory, Department of Livestock Services, Savar, Dhaka, Bangladesh; 2 Department of Pharmacology, Bangladesh Agricultural University, Mymensingh, Bangladesh; 3 Department of Gynecology, Obstetrics & Reproductive Health, Bangabandhu Sheikh Mujibur Rahman Agricultural University, Gazipur, Bangladesh; 4 National Institute of Food and Agriculture, Washington D. C. , United States; 5 Livestock Officer, Food and Agriculture Organization of the United Nations, Rome, Italy

**Keywords:** spirulina, arsenic, toxicity, reproductive parameters and rats

## Abstract

Spirulina (*Spirulina platensis*), has numerous health benefits including antioxidant, immunomodulatory, and anti-inflammatory activities, works against heavy metal toxicity, and is often used as a food supplement in human, animals, birds and fishes. This study aimed to evaluate the protective ability of the dietary spirulina against the toxic effects of inorganic arsenic (iAs) on male reproductive parameters in rats. Seventy-two mature Long-Evans male rats, dividing into six groups (T0, T1, T2, T3, T4 and T5) (12 rats/group) were included in this study. The T3, T4 and T5 group rats were treated with three consecutive doses (1.0 g, 1.5 g and 2.0 g/kg feed) of spirulina in feed along with 3.0 mg NaAsO_2_/kg body weight (BW) in drinking water (DW) daily for 90 days. Each rat of group T1 received NaAsO_2_ (3.0 mg/kg BW) in DW, and those of T2 group were fed with spirulina (2.0 g/kg feed) daily for 90 days. The rats of group T0 served as the control with normal feed and water. Total arsenic (tAs) contents, reproductive parameters (testicular weight, sperm motility and morphology), and histological changes in the testicles were evaluated in these rats. Arsenic dosing significantly (p=0.003, Kruskal-Wallis test) increased the tAs contents in the testicles, decreased testes weight, sperm morphology and motility compared to the controls. The effect of arsenic dosing was also evidenced by the histological changes like decreased germinal layers in the seminiferous tubules of the treated rats. Moreover, dietary spirulina (2.0 g/kg feed) supplementation significantly (p=0.011, Kruskal-Wallis test) lowered tAs contents in testicles and increases testes weights, sperm motility and morphology. Therefore, spirulina can be used as an effective dietary supplement to ameliorate the adverse effects of arsenic induced reproductive toxicities. However, further study is required to elucidate the underlying molecular mechanisms of reduction of arsenic induced reproductive toxicity by spirulina.

## Introduction

Arsenic (As) is an environmental toxicant, found in both organic and inorganic forms, which exists in air, natural water, soil, vegetation, plants, forests and marine products. This heavy metal affects both human and animals throughout the globe especially in Bangladesh and India (Maji et al., 2016). Humans and animals are generally exposed to As by consumption of contaminated ground water or through their food chains (Santra et al., 2013). Though liver and kidneys are most susceptible to As toxicity, As induced male reproductive toxicity leading to male infertility has drawn significant attention in the recent years (Sarkar et al., 2003; Souza et al., 2016a; Souza et al., 2016b; Souza et al., 2019; Zubair et al., 2017). Experimental exposure of As to male animals can cause a gradual accumulation of As in testicular tissues which in turn leads to the thickening of tubules basement membrane, vascular degeneration, hemorrhage in interstitial tissues, deformation of Leydig cells, absence of sperm bundles in some tubules, and significant reduction in sperm motility, sperm count and sperm abnormalities (Zubair et al., 2017; Zubair et al., 2014; Bashandy et al., 2016; Souza et al., 2019). Arsenic generates reactive oxygen species (ROS) by decreasing the activation of antioxidant enzymes, superoxide dismutase, catalase, and glutathione-s-transfrerase and glutathione peroxidase, thereby causing oxidative stress in the testis of rats (Manna et al., 2008; Lima et al., 2018; Singh et al., 2011) rats. Arsenic induction also caused increase in luminal areas with reduced accumulation of spermatozoa, necrotic changes with disarray in cellular organization (Aziz et al., 2018; Reddy et al., 2011). Although, various experimental models have been developed to understand how arsenic exposure causes these diverse disease outcomes, the actual molecular events resulting in reproductive and developmental toxicity from arsenic exposure remain unclear, and specific relationship between experimental and human exposures are not established yet (Prathima et al., 2018).

Cessation of drinking As contaminated water (Das and Sengupta, 2008; Pal et al., 2009) and ingesting As rich foods is the first step to control arsenicosis. Secondly, antioxidant supplement and feeding of As burden reducing agent seem to be beneficial for remedy of arsenicosis (Das and Sengupta, 2008; Khandker et al., 2006). Spirulina (*Spirulina platensis*), a blue-green alga, possesses antioxidant properties (Bashandy et al., 2016) with corrective properties against heavy metal toxicity, nephrotoxicity induced by heavy metals and drugs and also against cancer, tumor growth and malnutrition (Misbahuddin et al., 2006). Spirulina was also found to attenuate As-induced oxidative stress, testicular damage, and sperm abnormalities by its potent antioxidant activity and maintain the normalcy of the testicular architecture (Bashandy et al., 2016). Spirulina is a protective modulator of mercuric chloride-induced testicular injuries and oxidative stress (El-Desoky et al., 2013). Spirulina significantly lessen the increase in arsenic concentration, and the reduction in zinc concentration of testicular tissue resulted from sodium arsenite administration (Bashandy et al., 2016). Zinc acts as a cofactor for superoxide dismutase, preserves reduced glutathione, and induces metallothionein which has antioxidant and metal-chelating properties (Rooney, 2007), and also acts as an effective anti-inflammatory and antioxidant agent (Prasad et al., 2004). Spirulina is rich in protein with all essential amino acids, antioxidants, galaxy of phytonutrients and polysaccharides that trigger enzyme systems to enhance detoxification of As (Ahmad, 2001; El-Desoky et al., 2013). However, mitigation of As induced reproductive toxicity with spirulina using an animal model has not yet been extensively studied. Therefore, the present study evaluated the protective ability of the dietary spirulina against the toxic effects of iAs on the male reproductive parameters in rats.

## Material and methods

### Test chemicals

A 0.2% sodium arsenite (NaAsO_2_) (Merck, Darmstadt, Germany: 0.58 mg iAs/mg) stock solution was prepared with deionized water and preserved at 4 °C for use of maximum 7 days. Dried powder of spirulina (Sp) (Sigma Chemical Co., Ankara, Turkey) was obtained from the laboratory production at Arsenic Detection and Mitigation Laboratory, Department Pharmacology (ADM Lab), Bangladesh Agricultural University, Mymensingh, Bangladesh. Required amounts of the respective doses (1.0, 1.5 and 2.0g/kg feed) of the Sp were individually mixed with pellet feed and dried at 50 °C in an electric oven for at least 20 h. Then, the spirulina mixed feed was preserved in air tight polypropylene container for 5 days.

### Animals and experimental protocol

The experimental protocol of the current study was approved by the Institutional Review Board at the International Centre for Diarrhoeal Disease Research, Bangladesh (ICDDR’B) following review by the Research and Ethics Committees, and the Bangladesh Association for Laboratory Animal Sciences (BALAS), Bangladesh Agricultural University, Bangladesh under reference number 272/Pharma/2011dated 24 October 2011. A total of apparently healthy 72 adult male Long-Evans rats at the age of 5 to 6 months with an average body weight of 350.0 g were selected for the study. The rats were housed in sterilized polypropylene rat cages, in 12-h light–dark cycle, at an ambient temperature of 30 ± 2 °C and at a humidity of 70 ± 5% on a standard commercial pellet diet and drinking water (DW) *ad libitum*.

The rats were randomly divided into six equal groups (T0, T1, T2, T3, T4 and T5) comprising 12 rats in each group. The T1, T3, T4 and T5 group rats were individually fed with NaAsO_2_ (Merck, Darmstadt, Germany: 0.58 mg iAs/mg) at 3.0 mg/kg BW/day in DW. Concurrently, the rats of T3, T4 and T5 groups were individually supplemented with 3 doses of the spirulina (Sp) at 1.0, 1.5 and 2.0 g/kg feed, respectively in feed. Each rat of the T2 group received only spirulina supplementation (2.0 g/kg of feed) as Sp treated control group. The rats of the T1 group were fed only with 3.0 mg NaAsO_2_/kg BW/day in DW as iAs treated control while the rats of T0 group were supplied only normal feed and DW as non-treated control. The trial was continued up to 90 days.

In every morning, required amount of the prepared NaAsO_2_ solution (0.2%) for the rats of a group per day at the rate of 3 mg/kg BW/day was calculated and taken into an acid washed waterer. Then, a small amount of DW was added to the solution so as not to exceed the half of the daily requirement of DW for the rats of that particular group. This was done to ensure drinking of the total amount of NaAsO_2_ solution within 6 to 8 h of supply. After drinking the total amount of NaAsO_2_ solution, the rats were allowed to drink normal DW *ad libitum*. Parallel to the NaAsO_2_ feeding, the spirulina mixed feed with the respective doses were supplied *ad libitum* to the rats of respective groups*.*

### Sample collection

**Figure 1:**
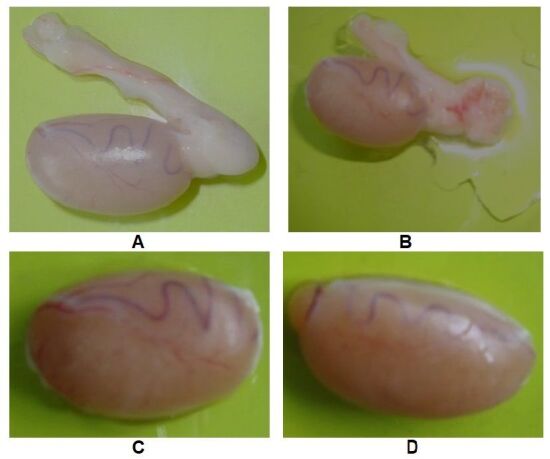
Photographs of rat testes and epididymis. (A) Rate testis with epididymis; (B) rat testis after incising the epididymis; (C) right testis without epididymis and (D) left testis without epididymis.

Samples were collected from the trial rats at 30 days interval starting from Day 0 (day before starting the treatment), Day 30, Day 60 and Day 90. From each animal group, 3 rats were randomly selected and sacrificed under ether anesthesia using sliding top chamber (Kent Scientific corporation) during sample collection (Bashandy et al., 2016). On each sampling day, testis and epididymis were immediately removed and dissected out (Figures 1A, B), and subjected to determination of reproductive parameters, quantitative measurement of total As (tAs) contents in each testis, and evaluation of histopathological changes in testicles. The collected testes were kept into phosphate buffered saline (PBS) in sterilized stopper glass vials and immediately transferred to a hot surface with 37 °C for determination of sperm motility and sperm morphology. After completion of the procedures for sperm motility and sperm morphology study, the testicles were individually weighed removing epididymis and other accessory parts (Figures 1C, D). Then, on each sampling day except on Day 90, one testicle was taken into individual polypropylene zipper bags and preserved at -20 °C until processed for determination of tAs contents and other testicle was discarded. On Day 90, after completion of reproductive parameters study, one testicle was collected for determination of tAs contents and the other testicle was taken into stopper glass vial filled with buffered neutral formalin (10 x sample volume) for histopathological examinations following washing with buffered neutral formalin and kept at room temperature until processed.

### Determination of reproductive parameters

#### Sperm motility, sperm morphology and testicular weight

The testicle in vials were kept on hot surface for about 5 min, each testicle was individually taken out of the vials and placed on a glass Petri dish. The epididymis from each testicle was dissected with an adequate longitudinal incision, which resulted in almost complete opening of the epididymis. Immediately after dissection, 100 to 200 µL of PBS heated at 37 ^0^C was poured on the opened part of the epididymis and another 100 to 200 µL of PBS was placed on the surface of the Petri dish at about 0.5 cm towards the dissected part of the epididymis connecting to the PBS placed on the epididymis. The color of the free part of the PBS became grayish after accumulation of sperm by swimming. About 10 µL of the grayish PBS was taken on a clean and dried glass slide and covered by a cover slip and the sperm motility was immediately studied under a microscope (Barkallah et al., 2020; El-Desoky et al., 2013) microscope.

Immediately after taking the sperm on the slide for examination of motility, the remaining sperm in the PBS on the Petri dish surface were taken into formol saline in individual Eppendorf tubes to study the sperm morphology and preserved at 4 ^0^C. The morphology of the sperm was studied using wet preparation technique under a Differential Interference Contrast (DIC) microscope (Olympus, Japan) fitted with a monitor and a digital camera. On each sampling day, after completing the sperm motility study and sperm collection for morphology study, the epididymis and other additional parts of the testicles were removed clearly with a sharp scissor keeping the testicles intact. The testicles were then individually soaked adequately on tissue paper and weighed separately with a calibrated electric balance.

### Determination of total arsenic contents in testicles

Total arsenic content (tAs) in each testicle was determined with an Atomic Absorption Spectrophotometer (AAS; model PG–990, PG Instruments Ltd., UK) coupled with a Flow Injection Hydride Generator (FI-HG). The instrument was controlled by a computer with AAWin software. The minimum detection limit of the instrument was 2.0 ppb of As. Following acid digestion and volume of samples, preparation of iAs standards and required analytical solutions, and reduction of samples, tAs contents in testicles were quantitatively determined. The accuracy and precision of tAs analyses in the samples were evaluated comparing with a commercially available certified reference material (CRM). The CRM reference number is GBW (E) 080684 and the reference concentration of total As was 0.11 ppm. The CRM was used in each set of digestion, reduction of samples and analysis of tAs and the recovery rate was recorded. When the recovery rate of CRM was observed less than 85% or above 100% in any set of the sample preparation process, the whole process (digestion to determination) was repeated (Afkhami-Ardakani et al., 2018; Barkallah et al., 2020).

### Histopathological examination

Histopathological procedures of testicles were carried out following standard procedure of tissue processing and sectioning. In brief, the testes were removed, cleaned of accessory tissue, weighted, and fixed. Fragments of testis were immersed in alcoholic formalin fixative solution for 24 h, dehydrated in ethanol series, and embedded in paraffin. Sections of 3 µm thickness were obtained using rotary microtome, and stained with Hematoxylin and Eosin. Histological sections-stained tissue sections were examined under DIC microscope (Olympus, Japan) fitted with a monitor and a digital camera (Afkhami-Ardakani et al., 2018; Barkallah et al., 2020). The mean seminiferous tubule diameter was obtained by randomly measuring 30 tubular cross sections, as circular as possible. These sections were also used to measure the epithelium height and luminal diameter, according to previously published methods (Souza et al., 2016b).

### Statistical analysis

Descriptive data analysis was performed using the FREQ procedure of SAS 9.4 (SAS Inst. Inc., Cary, NC, USA). The data were expressed as mean ± SD (Standard deviation). Each set of data (mean ± SD, n=12 for each group) was tested for normality (Shapiro-Wilk test) and homogeneity of variance. Means were compared and analyzed by two-way analysis of variance (ANOVA) followed by a non-parametric test (Kruskal-Wallis test) to reveal the mean differences among the parameters tested (Hoque et al., 2019; Hoque et al., 2020). Least Significance Difference (LSD) was calculated to compare the variations between treatments where ANOVA showed significant differences. Differences among the groups were considered significant at p < 0.05 level.

## Results

### Spirulina overcomes testicular weights reduction in arsenic-induced rats

Inorganic arsenic (iAs) induction (T1) significantly decreased right testes weights compared to control (T0) and spirulina (Sp) treatment (T3, T4 and T5) prevented the iAs induced weight loss in the trial rats. All of the Sp treated groups (with and without iAs dosing) significantly increased the right testes weights of the trial rats compared to control (T0) and iAs-group (T1). However, the right testes weights did not differ significantly on Day 30 while that varied significantly on Day 60 (p=0.029, Kruskal-Wallis test) and on Day 90 (p=0.021, Kruskal-Wallis test) among the trial groups (Table 1). The highest weights were found in the rats of the T5 group (Sp: 2.0 g/kg feed) and the lowest in that of the T3 group (Sp: 1.0 g/kg feed) among the iAs induced Sp treated groups. The average efficacy of the Sp treatment was improved (10.82% and 49.41% increase compared to T0 and T1, respectively) and the highest doses of the Sp (T5: 15.89% and 56.25% compared to T0 and T1, respectively) were found the best among the doses of the Sp in increasing the right testes weights at the end of the trial.

**Table 1:** Testes weight trial rats. Right, left and paired testes weights on sampling days (Day 0, 30, 60 and 90). Number of animals, n= 12/group.

***Groups of animal***	**Right testes weights (g)**						
	***Day 0***	***Day 30***	***Day 60***		***Day 90***		
Control group (T0)	1.39±0.12	1.41±0.20	1.27±0.18^b^		1.51±0.04^a^		
As-group (T1) (% compared to T0 values)	1.37±0.09	1.20±0.2 (- 14.89)	1.06±0.06^c^ (- 16.54)		1.12±0.05^b^ (- 25.83)		
Sp-group (T2) (% compared to T0 values)	1.38±0.10	1.54±0.28 (9.22)	1.62±0.23^a^ (27.56)		1.53±0.07^a^ (1.32)		
As plus Sp group (T3) (% compared to T0; and to T1)	1.39±0.03	1.47±0.09 (4.26; 22.50)	1.52±0.03^a^ (19.69; 43.40)		1.60±0.08^a^ (5.96; 42.86)		
As plus Sp group (T4) (% compared to T0; and to T1)	1.38±0.05	1.60±0.07 (13.48; 33.33)	1.63±0.05^a^ (28.35; 53.77)		1.67±0.02^a^ (10.60; 49.11)		
As plus Sp group (T5) (% compared to T0; and to T1)	1.35±0.03	1.61±0.08 (14.18; 34.17)	1.69±0.05^a^ (33.07; 59.43)		1.75±0.06^a^ (15.89; 56.25)		
*LSD*	ND	-	0.12		0.21		
*Level of significance*	ND	NS	**		**		
**Left testes weights (g)**							
Control group (T0)	1.26±0.15	1.29±0.17		1.20±0.14^b^		1.38±0.04	
As-group (T1) (% compared to T0 values)	1.22±0.07	1.10±0.21 (- 14.73)		0.97±0.08^c^ (- 19.17)		1.05±0.05 (- 23.91)	
Sp-group (T2) (% compared to T0 values)	1.27±0.11	1.37±0.23 (6.20)		1.49±0.13^a^ (24.17)		1.46±0.07 (5.80)	
As plus Sp group (T3) (% compared to T0; and to T1)	1.27±0.04	1.37±0.13 (6.29; 24.55)		1.47±0.05^a^ (22.50; 51.55)		1.52±0.07 (10.14; 44.76)	
As plus Sp group (T4) (% compared to T0; and to T1)	1.27±0.06	1.39±0.10 (7.75; 26.36)		1.54±0.04^a^ (28.33; 58.76)		1.56±0.02 (13.04; 48.57)	
As plus Sp group (T5) (% compared to T0; and to T1)	1.23±0.04	1.48±0.06 (14.73; 34.55)		1.56±0.06^a^ (30.00; 60.82)		1.65±0.05 (19.57; 57.14)	
*LSD*	ND	-		0.09		-	
*Level of significance*	ND	NS		**		NS	
**Paired testes weight (g)**							
Control group (T0)	2.66±0.26	2.71±0.37	2.47±0.32^b^			2.90±0.08^b^	
As-group (T1) (% compared to T0 values)	2.59±0.15	2.30±0.43 (- 15.13)	2.03±0.15^c^ (- 17.81)			2.17±0.09^c^ (- 25.17)	
Sp-group (T2) (% compared to T0 values)	2.65±0.21	2.91±0.51 (7.38)	3.11±0.37^a^ (25.91)			3.00±0.13^ab^ (3.45)	
As plus Sp group (T3) (% compared to T0; and to T1)	2.66±0.08	2.84±0.22 (4.80; 23.48)	2.99±0.08^ab^ (21.05; 47.29)			3.13±0.15^a^ (7.93; 44.24)	
As plus Sp group (T4) (% compared to T0; and to T1)	2.65±0.10	2.99±0.14 (10.33; 30.00)	3.18±0.09^a^ (28.74; 56.65)			3.23±0.04^a^ (11.38; 48.85)	
As plus Sp group (T5) (% compared to T0; and to T1)	2.58±0.06	3.09±0.04 (14.02; 34.35)	3.25±0.10^a^ (31.58; 60.10)			3.40±0.11^a^ (17.24; 56.68)	
*LSD*	ND	-	0.22			0.22	
*Level of significance*	ND	NS	**			**	

Data were presented as mean ± SD; Values within the parenthesis indicate percentage; Values within the parenthesis without any sign indicates increased percentage; Values within the parenthesis with ‘—‘ sign indicates decreased percentage value. ND: Analysis not done; NS: Not significant. ** Significant at 1% level of probability. In a column values with similar superscript or without superscript do not differ significantly while values with dissimilar superscript differ significantly (as per DMRT).

Similar to the right testis, iAs induction (T1) significantly decreased the left testes weights compared to the control (T0) and Sp treatment (T3, T4 and T5) prevented the iAs induced weight loss in the trial rats. All of the doses of the Sp (T3, T4 and T5) and Sp treated control (T2) increased the left testes weights of the trial rats compared to control (T0) and iAs-group (T1). However, the left testes weights of the trial rats did not differ significantly on Day 30 and Day 90 while that varied significantly on Day 60 (p=0.037, Kruskal-Wallis test) among the trial groups (Table 1). Hence, the dietary Sp was found effective (Sp: average 14.25% vs.50.16% increased compare to T0 vs. to T1) and the highest doses of the Sp (T5: 19.57% vs. 57.14% compare to T0 vs. to T1) were found best among the Sp treated groups in increasing the left testes weights in the iAs induced rats at the end of trial (Table 1).

In consistent with right and left testes weights, the paired testes weights of the trial rats significantly (p=0.022, Kruskal-Wallis test) decreased due to iAs induction (T1) compared to control (T0) and the Sp feeding successfully prevented the weight loss compared to control (T0) and iAs-group (T1). The paired testes weights did not vary significantly on Day 30 and Day 90 while that differed significantly on Day 60 (p=0.013, Kruskal-Wallis test) among the trial groups (Table 1). The paired testes weights of the rats were highest in the T5 while that was lowest in the T1 group on all of the sampling days after starting the treatment. All the doses of the Sp increased the paired testes weights compared to T1 group. However, the average efficacy of the Sp was increased (12.18% and 49.92% compare to T0 and to T1, respectively) and the highest dose of the Sp (T5) was increased the efficacy 17.24% and 56.68% compare to T0 and to T1, respectively and found best among the doses of the Sp in increasing the paired testes weights at the end of the trial (Table 1).

### Spirulina increases sperm motility in arsenic-induced rats

The sperm motility of the trial rats significantly reduced after iAs induction (T1) compared to control (T0) and Sp treatment significantly increased the motility at the end of the trial compared to T1 and maintain the motility above or near the level of the T0. However, the sperm motility of the rats did not differ significantly on Day 30 and Day 60 but that varied significantly on Day 90 (p=0.023, Kruskal-Wallis test) among the trial groups (Table 2). The Sp treatment increased (average 11.80%) the motility and the highest dose of the Sp (T5: 16.66%increased) were observed best among the doses of Sp in increasing sperm motility in the iAs induced rats compared to the T1 on Day 90. The highest (T5) and the intermediate (T4) doses of Sp increased the sperm motility above and at the level of T0 at the end of the trial (Table 2).

**Table 2:** Sperm motility of trial rats on sampling days (Day 0, 30, 60 and 90). Number of animals, n= 12/group.

**Groups of animals**	**Sperm motility (%)**			
	**Day 0**	**Day 30**	**Day 60**	**Day 90**
Control group (T0)	90.00±5.00	88.33±2.89	91.67±2.89	90.00±5.00^ab^
As-group (T1) (% compared to T0 values)	90.00±5.00	83.33±2.89 (- 5.66)	81.67±5.77 (- 10.91)	80.00±0.00^c^ (- 11.11)
Sp-group (T2) (% compared to T0 values)	88.33±5.77	86.67±2.89 (- 1.88)	88.33±2.89 (- 3.64)	91.67±2.89^ab^ (1.86)
As plus Sp group (T3) (% compared to T0; and to T1)	91.67±2.89	81.67±2.89 (-7.54; - 1.99)	83.33±7.64 (- 9.10; 2.03)	85.00±5.00^b^ (- 5.56; 6.25)
As plus Sp group (T4) (% compared to T0; and to T1)	90.00±5.00	85.00±5.00 (- 3.77; 2.00)	86.67±2.89 (- 5.45; 6.12)	90.00±5.00^ab^ (0.00; 12.50)
As plus Sp group (T5) (% compared to T0; and to T1)	91.67±5.77	88.33±5.77 (0.00; 6.00)	90.00±5.00 (- 1.82; 10.20)	93.33±2.89^a^ (3.70; 16.66)
*LSD*	ND	-	-	3.421
*Level of significance*	ND	NS	NS	**

Data were presented as mean ± SD; Values within the parenthesis indicate percentage value; Values within the parenthesis without any sign indicates increased percentage value; Values within the parenthesis with ‘—‘sign indicates decreased percentage value. ND: Analysis not done; NS: Not significant. ** Significant at 1% level of probability. In a column values with similar superscript or without superscript do not differ significantly while values with dissimilar superscript differ significantly (as per DMRT).

### Spirulina improves sperm morphology in arsenic-induced rats

**Figure 2:**
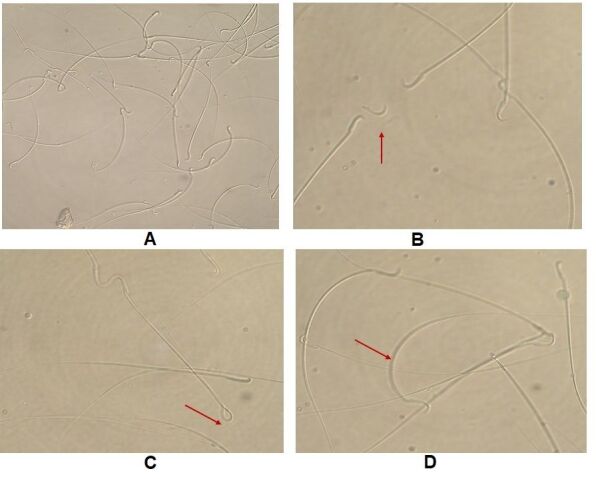
Sperm morphology of trial rats on sampling days. (A) Photograph of rat spermatozoa ×200; (B) rat sperm with detached tail in trial rats on sampling days ×400; (C) rat sperms with coiled tail in trial rats on sampling days ×400; (D) rat sperms with bent tail in trial rats on sampling days ×400. Arrow indicate the specific sperm abnormality.

Sperm of the trial rats (Figure 2A) were observed with detached tail (Figure 2B), coiled tail (Figure 2C) and with bent tail (Figure 2D) during the morphology study of the sperm.

The sperm with detached tail of the rats showed the highest number in the T4 while the lowest numbers (nil value) were in the T1, and T3 groups on Day 0. The highest numbers were observed in the T1 group on other sampling days while the lowest number was found in the T2 and T3 on Day 30 (based on percent values), in the T4 on Day 60, and in the T2 group on Day 90 (nil value). However, the numbers of the sperm with detached tail of the rats varied significantly (p=0.039, Kruskal-Wallis test) among the trial groups on all the data analyzed sampling days. The numbers of the sperm with detached tail did not differ significantly among all of the groups on Day 30 and Day 60, except the T0 and T1 on Day 30, and except the T0, T1 and T3 on Day 60. Similarly, the numbers on Day 90 did not vary significantly between the T2 and T3 and among the iAs plus Sp group (Table 3). Therefore, it showed (based on percent values) that the iAs induction significantly increased the numbers of sperm with detached tail in the rats compared to the control while the Sp treatment (with and without iAs) significantly decreased that compared to the T1 and the T0 groups on all of the data analyzed sampling days. The average efficacies of the Sp (74.9% vs. 87.67% compare to T0 vs. to T1) and all their doses (74.10 to 75.90% vs. 87.28 to 88.17% compare to T0 vs. to T1) were found almost similarly effective in reducing the number of the sperm with detached tail in the iAs induced rats (Table 3).

**Table 3:** Sperm morphology: Number of sperm with detached tail in trial rats on sampling days (Day 0, 30, 60 and 90). Number of animals, n= 12/group.

**Groups of Animal**		**Day 0**				**Day 30**				**Day 60**				**Day 90**	
		**Sperm counted**		**Sperm with detached tail**		**Sperm counted**		**Sperm with detached tail**		**Sperm counted**		**Sperm with detached tail**		**Sperm counted**	**Sperm with detached tail**
Control group (T0)	1069.00±165.01		4.33(0.40)±1.53		1233.00±78.71		9.00(0.73) ±3.00^b^		1130.67±214.97		15.00(1.33)±3.00^b^		1144.67±82.23		19.00(1.66)±2.65^b^
As-group (T1) (% compared to T0 values)	1161.67±147.96		0.00(0.00)±0.00		1134.67±78.65		22.33(1.96)±3.06^a^ (168.49)		1139.33±77.78		32.67(2.86)±4.16^a^ (115.04)		1168.33±125.25		39.67(3.38)±8.14^a^ (103.61)
Sp-group (T2) (% compared to T0 values)	1134.67±103.80		4.33(0.38)±1.53		1066.67±83.51		3.67(0.34)±2.08^c^ (- 53.42)		1097.67±86.74		4.00(0.36)±1.00^d^ (- 72.93)		1093.00±23.26		0.00(0.00)±0.00^d^ (- 100.00)
As plus Sp group (T3) (% compared to T0; and to T1)	1104.67±23.54		0.00(0.00)±0.00		1133.67±77.73		3.67(0.33)±1.53^c^ (- 54.79; - 83.16)		1107.67±96.40		8.67(0.79)±1.53^c^ (- 40.60; - 72.38)		1077.67±120.98		4.67(0.42)±2.52^c^ (- 74.70; - 87.57)
As plus Sp group (T4) (% compared to T0; and to T1)	1116.67±92.39		5.00(0.45)±3.00		1059.67±101.11		4.00(0.37)±1.00^c^ (- 49.32; - 81.12)		1098.33±48.23		3.33(0.30)±1.53^d^ (- 77.44; - 89.51)		1113.33±97.22		4.33(0.40)±1.53^c^ (- 75.90; - 88.17)
As plus Sp group (T5) (% compared to T0; and to T1)	1062.33±77.02		4.33(0.41)±1.53		1091.67±69.62		4.33(0.40)±1.53^c^ (- 45.21; - 79.59)		1161.00±95.92		4.00(0.34)±1.00^d^ (- 74.44; - 88.11)		1080.33±48.69		4.67(0.43)±0.58^c^ (- 74.10; - 87.28)
*LSD*	ND		ND		ND		1.784		ND		1.92		ND		2.933
*Level of significance*	ND		ND		ND		**		ND		**		ND		**

Data were presented as mean ± SD; Values within the parenthesis prefixing SD indicate values in percent in respect to the counted sperm; Values within parenthesis suffixing SD without any sign indicates increased percentage value and values within the parenthesis suffixing SD with ‘—‘ sign indicates decreased percentage value based on the percentage value mentioned prefixing SD. ND: Analysis not done. ** Significant at 1% level of probability. In a column values with similar superscript or without superscript do not differ significantly whereas values with dissimilar superscript differ significantly (as per DMRT).

The highest number of sperm with coiled tail of the rats was observed in the T3 group and the lowest numbers (nil value) were found in the T0, T1, T2 and T5 groups on Day 0 while the numbers were found highest in the T1 on other sampling days while that were lowest in the T0, T3 and T4 on Day 30, in the T4 on Day 60, and in the T0 and T3 on day 90 (Table 4). However, the numbers of sperm with coiled tail of the rats significantly varied (p=0.044, Kruskal-Wallis test) among the trial groups on all of the data analyzed sampling days. The numbers of the sperm with coiled tail were significantly increased in the trial rats due to iAs induction (T1) compared to the control (T0) while that were significantly decreased with Sp treatment (with and without iAs) compared to the T1 group on all of the data analyzed sampling days. The data showed (based on percent values) that the numbers of the sperm with coiled tail of the iAs induced trial rats were reduced with Sp treatments at the control levels in the T3, and T4 on Day 30; in the T3, T4 and T5 on Day 60; and only in the T3 on Day 90. However, the average efficacies of the Sp (90.87% reduced) were varied significantly in reducing the number of sperm with coiled tail among the treated groups during the trial periods, and the lowest dose (1 g/kg feed) of the Sp was found most effective (100.0% reduced) in the iAs induced rats compared to T1 on Day 90 (Table 4).

**Table 4:** Sperm morphology: Number of sperm with coiled tail in trial rats on sampling days (Day 0, 30, 60 and 90). Number of animals, n= 12/group.

**Groups of Animal**	**Day 0**		**Day 30**		**Day 60**		**Day 90**	
	**Sperm counted**	**Sperm with coiled tail**	**Sperm counted**	**Sperm with coiled tail**	**Sperm counted**	**Sperm with coiled tail**	**Sperm counted**	**Sperm with coiled tail**
Control group (T0)	1069.00±165.01	0.00(0.00)±0.00	1233.00±78.71	0.00(0.00)±0.00^c^	1130.67±214.97	3.00(0.26)±1.00^b^	1144.67±82.23	0.00(0.00)±0.00^c^
As-group (T1) (% compared to T0 values)	1161.67±147.96	0.00(0.00)±0.00	1134.67±78.65	7.33(0.65)±1.53^a^ (0/0)†	1139.33±77.78	16.33(1.42)±4.16^a^ (446.15)	1168.33±125.25	32.67(2.78)±6.03^a^ (0/0)†
Sp-group (T2) (% compared to T0 values)	1134.67±103.80	0.00(0.00)±0.00	1066.67±83.51	3.67(0.35)±1.53^b^ (0/0)†	1097.67±86.74	4.33(0.39)±1.15^b^ (50.00)	1093.00±23.26	4.00(0.37)±1.00^bc^ (0/0)†
As plus Sp group (T3) (% compared to T0; and to T1)	1104.67±23.54	4.67(0.42)±2.52	1133.67±77.73	0.00(0.00)±0.00^c^ [(0/0)†; - 100.00]	1107.67±96.40	4.67(0.42)±0.58^b^ (61.54; - 70.42)	1077.67±120.98	0.00(0.00)±0.00^c^ [(0/0)†; - 100.00]
As plus Sp group (T4) (% compared to T0; and to T1)	1116.67±92.39	4.00(0.36)±1.73	1059.67±101.11	0.00(0.00)±0.00^c^ [(0/0)†; - 100.00]	1098.33±48.23	0.00(0.00)±0.00^c^ (- 100.00; - 100.00)	1113.33±97.22	4.67(0.42)±2.08^b^ [(0/0)†; - 84.89]
As plus Sp group (T5) (% compared to T0; and to T1)	1062.33±77.02	0.00(0.00)±0.00	1091.67±69.62	5.00(0.46)±2.00^ab^ [(0/0)†; - 29.23]	1161.00±95.92	5.00(0.43)±1.00^b^ (65.38; - 69.72)	1080.33±48.69	3.67(0.34)±1.15^bc^ [(0/0)†; - 87.77]
*LSD*	ND	ND	ND	1.441	ND	1.58	ND	2.176
*Level of significance*	ND	ND	ND	**	ND	**	ND	**

Data were presented as mean ± SD; Values within the parenthesis prefixing SD indicate values in percent in respect to the counted sperm; Values within parenthesis suffixing SD without any sign indicates increased percentage value and Values within the parenthesis suffixing SD with ‘—‘ sign indicates decreased percentage value based on the percentage value mentioned prefixing SD. ND: Analysis not done. ** Significant at 1% level of probability. In a column values with similar superscript or without superscript do not differ significantly whereas values with dissimilar superscript differ significantly (as per DMRT).

The numbers of sperm with bent tail of the rats were found highest in the T2 and that in the T0 showed the lowest numbers on Day 0 while the iAs-group (T1) had the highest numbers on other sampling days and the lowest numbers were in the T5 on Day 30 and Day 90, and in the T0 group on Day 60. On Day 0, the numbers of sperm with bent tail were found lower in majority of the groups than that on other sampling days and almost at similar level in all the groups (Table 5). Similar to other two morphological parameters of sperm, the numbers of sperm with bent tail showed significant difference (p=0.002, Kruskal-Wallis test) among the trial groups on all of the data analyzed sampling days. The numbers of the sperm with bent tail were significantly increased in the trial rats due to iAs induction (T1) compared to the control while that were significantly decreased with Sp treatment (with and without iAs) compared to the T1 on all of the data analyzed sampling days. The data showed (based on percent values) that the numbers of the sperm with bent tail of the trial rats were reduced at the control level with all the doses of the Sp. Hence, the average efficacy of the Sp was found (42.89% vs. 78.80% decreased compare to T0 vs. to T1), and the highest (T5) doses of the Sp (T5: 53.21% vs. 82.63%; decreased compare to T0 vs. to T1) were found best in reducing the number of the sperm with bent tail in the iAs induced rats at the end of the trial (Table 5).

**Table 5:** Sperm morphology: Number of sperm with bent tail in rats after As, spirulina and As plus spirulina treatments. Number of animals, n= 12/group.

**Groups of Animal**	**Day 0**		**Day 30**		**Day 60**		**Day 90**	
	**Sperm counted**	**Sperm with bent tail**	**Sperm counted**	**Sperm with bent tail**	**Sperm counted**	**Sperm with bent tail**	**Sperm counted**	**Sperm with bent tail**
Control group (T0)	1069.00±165.01	19.33(1. 80)±3.51	1233.00±78.71	16.33(1.32)±2.08^d^	1130.67±214.97	18.00(1.61)±2.65^d^	1144. 67±82.23	30.33(2.65)±3.06^b^
As group (T1) (% compared to T0 values)	1161.67±147.96	20.33(1.76)±1.53	1134.67±78.65	70.33(6.21)±3.06^a^ (370.45)	1139.33±77.78	76.00(6.67)±8.72^a^ (314.45)	1168.33±125.25	82.67(7.14)±4.73^a^ (169.43)
Sp-group (T2) (% compared to T0 values)	1134.67±103.80	22.67(2.00)±3.51	1066.67±83.51	50.67(4.73)±9.07^b^ (258.33)	1097.67±86.74	33.00(3.03)±2.00^bc^ (88.20)	1093.00±23.26	18.00(1.65)±2.00^c^ (- 37.74)
As plus Sp group (T3) (% compared to T0; and to T1)	1104.67±23.54	20.00(1.82)±3.61	1133.67±77.73	41.00(3.62)±3.00^c^ (174.24; - 41.71)	1107.67±96.40	29.33(2.67)±3.51^c^ (65.84; - 59.97)	1077.67±120.98	18.33(1.70)±2.52^c^ (- 35.85; - 76.19)
As plus Sp group (T4) (% compared to T0; and to T1)	1116.67±92.39	21.33(1.94)±5.03	1059.67±101.11	40.33(3.87)±6.66^c^ (193.18; - 37.68)	1098.33±48.23	30.00(2.74)±3.61^c^ (70.19; - 58.92)	1113.33±97.22	17.67(1.60)±2.08^c^ (- 39.62; - 77.59)
As plus Sp group (T5) (% compared to T0; and to T1)	1062.33±77.02	20.00(1.87)±4.36	1091.67±69.62	20.67(1.90)±2.08^d^ (43.94; - 69.40)	1161.00±95.92	38.33(3.30)±4.16^b^ (104.97; - 50.52)	1080.33±48.69	13.33(1.24)±1.53^c^ (- 53.21; - 82.63)
*LSD*	ND	ND	ND	4.462	ND	4.42	ND	2.745
*Level of significance*	ND	ND	ND	**	ND	**	ND	**

Data were presented as mean ± SD; Values within the parenthesis prefixing SD indicate values in percent in respect to the counted sperm; Values within parenthesis suffixing SD without any sign indicates increased percentage value and values within the parenthesis suffixing SD with ‘—‘sign indicates decreased percentage value based on the percentage value mentioned prefixing SD. ND: Analysis not done. ** Significant at 1% level of probability. In a column value with similar superscript or without superscript do not differ significantly whereas values with dissimilar superscript differ significantly (as per DMRT).

### Spirulina reduces arsenic contents during arsenic-induced toxicities in male rats

The testicle of the rats contained the highest tAs in the T0 group on Day 0 and in the T1 on the remaining sampling days while the lowest contents were found in the T5 on Day 0, in the T0 group on Day 30, Day 60 and Day 90. On Day 0, the tAs contents of the testicle of almost all the trial groups were much lower than that on other sampling days. It is evident that almost all of the doses of the spirulina reduced the tAs contents of the testicle of the iAs induced rats compared to the T1 on all the data analyzed sampling days. The Sp treatment decreased tAs contents by average 40.08% and the highest (T5) dose of the Sp (49.25% decreased) showed best efficacy among the doses of the Sp in reducing the tAs contents from testicle of the iAs induced trial rats compared to the T1 group at the end of the trial. The data showed that the tAs contents of the testicle of the rats varied significantly (p=0.003, Kruskal-Wallis test) among the trial groups on all the data analyzed sampling days, although that did not differ significantly among the T0 and T2 on Day 30 and Day 60, respectively. All the doses of Sp on all the data analyzed sampling days, except the lowest dose of the Sp (T3) on Day 30; significantly (p=0.003, Kruskal-Wallis test) reduced the tAs contents compared to the T1 group. However, the tAs contents did not differ significantly among the T3, T4 and T5 on Day 30 and between the T4 and T5 on Day 60 and Day 90 (Table S1).

### Spirulina recuperates germinal epithelial layer depletion during arsenic-induced toxicities

**Figure 3:**
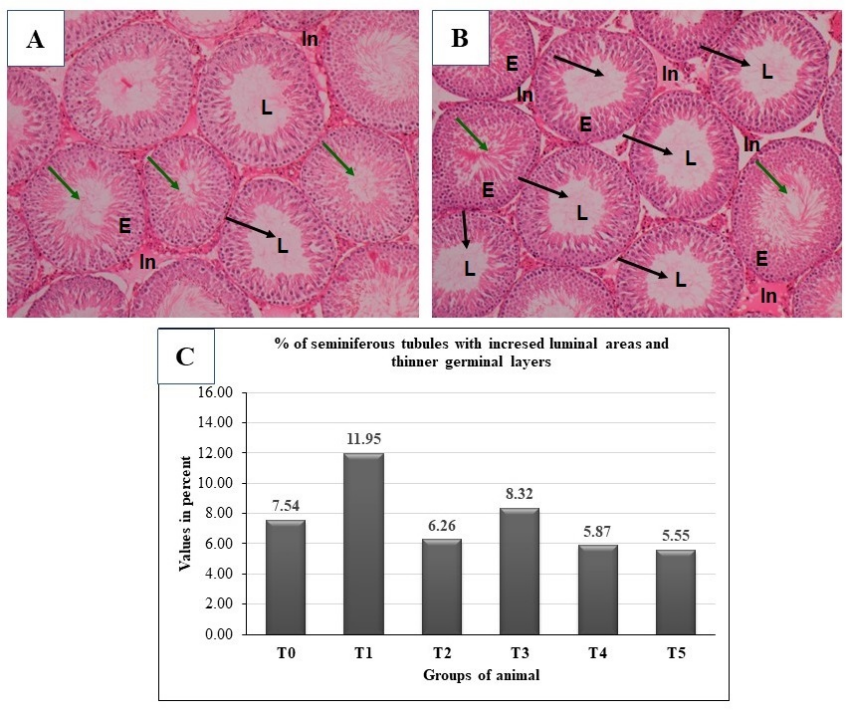
Hematoxylin and eosin-stained histopathological changes of testis in experimental and control groups. (A) Histopathological changes in testicles of control rat x1000; (B) histopathological changes in testicles of iAs induced control rats x1000; Green arrows indicate normal seminiferous tubules and black arrows indicate seminiferous tubules with increased luminal areas and thinner germinal layers; and (C) percent of the seminiferous tubules with increased luminal areas and thinner germinal layers in the testicle of trial rats on sampling days. Number of animals, n= 12/group.

Arsenic induction (T1) caused remarkable histopathological changes in the testicles of the trial rats, which include seminiferous tubules with increased luminal areas with reduced accumulation of spermatozoa, thinner germinal layers with markedly decreased spermatogenic cell population and with disarray in cellular organization, thicker tubule basement membrane and absence of sperm bundles in some tubules compared to the T0 (Figures 3A, B).The highest number of the seminiferous tubules with increased luminal areas and thinner germinal layers was found in the rats of the T1 while the lowest number was found in that of T5 group. The numbers of these changes varied significantly (p = 0.042, Kruskal-Wallis test) among the rats of the trial groups. However, the numbers in the rats did not significantly differ between the T4 and T5 groups (Table 6; Figure 3C). The data (based on percent values) showed that only the highest (T5) and intermediate doses (T4) of the Sp reduced the numbers of the seminiferous tubules with increased luminal areas and thinner germinal layers at the control level. Finally, the Sp treatment reduced the numbers of seminiferous tubules with increased luminal areas and thinner germinal layers in the testicle of the iAs induced rats by average 12.73% vs. 44.94% compared to T0 vs. to T1 and the highest doses of Sp (T5: 26.39% vs. 53.56% compared to T0 vs. to T1) was found best among the doses of the Sp (Table 6; Figure 3C).

**Table 6:** Incidence of seminiferous tubules with increased luminal areas and thinner germinal layers in the testicle of trial rats on sampling days. Number of animals, n= 12/group.

**Groups of Animal**	**Seminiferous tubules counted**	**Seminiferous tubules with increased luminal areas and thinner germinal layers in the testicle**
Control group (T0)	159.00±6.24	12.00(7.54)±1.00^b^
As-group (T1) (% compared to T0 values)	144.33±8.50	17.33(11.95)±3.51^a^ (58.49)
Sp-group (T2) (% compared to T0 values)	159.67±15.04	10.00(6.26)±1.00^b^ (- 16.98)
As plus L-Sp group-I (T3) (% compared to T0; and to T1)	140.67±10.97	11.67(8.32)±0.58^b^ (10.34; - 30.38)
As plus L-Sp group-II (T4) (% compared to T0; and to T1)	137.00±13.89	8.00(5.87)±1.00^c^ (- 22.15; - 50.88)
As plus L-Sp group-III (T5) (% compared to T0; and to T1)	139.00±11.36	7.67(5.55)±0.58^c^ (- 26.39; - 53.56)
*LSD*	ND	1.631
*Level of significance*	ND	**

Figures indicate mean ± SD; Figures within the parenthesis prefixing SD indicate values in percent in respect to the counted seminiferous tubules; Figures within the parenthesis suffixing SD without any sign indicates increased percentage value and figures within the parenthesissuffixing SD with ‘—‘ signindicates decreased percentage value based on the percentage value mentioned prefixing SD. ND: Analysis not done. ** Significant at 1% level of probability. In a column figures with similar superscript or without superscript do not differ significantly whereas figures with dissimilar superscript differ significantly (as per DMRT).

## Discussion

This study provides evidence for the first time that dietary supplementation of Sp at a lower concentration of 2 g/kg feed could ameliorate iAs-induced reproductive toxicities in male rats while previous studies (Ahmed et al., 2019; Bashandy et al., 2016; Sadek et al., 2017; Barkallah et al., 2020) reported that Sp at 3-5 g/kg feed could mitigate hazardous effects of iAs toxicity. In our study, iAs was given in drinking water (DW) at the dose rate of 3.0 mg/kg BW/day which produced a varied degree of reproductive toxicity. Sodium arsenate and sodium arsenite administered in rat diet at doses up to 400 and 250 µg/g arsenic, respectively, were negative in 2-year bioassays performed in rats. Administration of arsenite with a dose rate of 5 mg/kg body weight to rats (Jana et al., 2006; Ahmed et al., 2019) and Teddy goat bucks (Zubair et al., 2020) showed significant reduction in the level of testosterone, key hormone for the regulation of spermatogenesis and sexual behavior in the male reproductive system. Arsenite is more deleterious to male fertility than arsenate, and exposure to 10 mg/L arsenite reduced daily sperm production via H2O2 overproduction and germ cells loss in male Wistar rats (Lima et al., 2018). In another study, arsenic tri oxide at 3 mg /kg body wt/day in a single dose for 28 consecutive days to male Wistar rats caused increase in seminiferous tubular luminal size coupled with reduced accumulation of spermatozoa, and signs of necrotic changes with disarray in cellular organization (Mukherjee and Mukhopadhyay, 2009). Several previous reports suggested that adult rats imbibing 10 mg/L sodium arsenate or 0.01 mg/L sodium arsenite were compensated for by damage to male reproductive functions, suggesting that adverse effects on fertility might be occurring with this dose rates (Souza et al., 2016a; Medeiros et al., 2019). Thus, the dose rate of iAs we used in the present study is supported by most of the previous studies (even much lower), and findings also corroborate with that of previous studies.

Our data regarding reproductive parameters show almost similar scenarios in the right and left testes weights, and paired testes weights (Table 1), and as the paired testicular weight is considered as a valuable index of reproductive toxicity in male animals (Amann, 1981; Bashandy et al., 2016; El-Desoky et al., 2013). The paired testicular weights became lowest following iAs dosing alone to the trial rats of all groups on sampling days (Day 0, 30, 60 and 90) suggesting that iAs feeding can decrease the testes weights in rats compared to the control (T0) rats. The rats treated with the spirulina (Sp) alone (T2) significantly increased the paired testes weights compared to those of T0, T1 and T3 groups. This finding indicates that the Sp feeding to the rats improved the paired testes weights as also reported several earlier studies (Bashandy et al., 2016; Yousef et al., 2003; Rezvanfar et al., 2008). Similarly, the paired testes weights in both As and spirulina treated groups were increased gradually up to Day 90. Administration of Sp (1.0, 1.5 and 2.0 g/kg feed) significantly inhibited the iAs-induced reductions in testes weights. The average efficacy of the Sp was increased by 12.18% and 49.92% compared to control (T0) and iAs induced group (T1). Compared to 1.0 and 1.5 g/kg feed Sp dosing, 2 g/kg Sp supplementation in feed significantly ameliorate the reductions in testes weights induced by iAs. The effect of Sp dosing was particularly evident in the group T5 rats which had the highest paired testes weights suggesting that increase dose (2.0 g/kg feed) of Sp can effectively ameliorate iAs induced reproductive toxicity (Bashandy et al., 2016; Jana et al., 2006; Afkhami-Ardakani et al., 2018). Similar to the present findings, several earlier studies reported that the paired testicular weights were significantly decreased after iAs induction in rats (Jana et al., 2006; El-Desoky et al., 2013), and the paired testicular mass was also found to be decreased in iAs-treated animals and this decrease in testicular mass was consistent with elimination of germ cells (Afkhami-Ardakani et al., 2018).

It was also observed that iAs reduces epididymal sperm motility significantly. The decreased sperm motility may be elucidated by the increased oxidative stress, as spermatozoa are particularly susceptible to oxidative stress (Afkhami-Ardakani et al., 2018). According to a previous report, Sp protects sperm against oxidative stress induced by arsenic (Afkhami-Ardakani et al., 2018). In this study, administration of Sp (10, 1.5 and 2.0 g/kg feed) iAs-treated rats prevented the iAs-induced deteriorations in sperm motility. The results of the current study revealed that the sperm motility of the iAs-induced rats decreased gradually from Day 30 up to Day 90, and always remained lower (80 to ≥83%) compared to the control (≥ 88 to 90%) across the sampling days. This finding indicates that iAs dosing alone reduced the sperm motility and corroborated with the finding of Chiou et al. who reported that arsenic (As_2_O_3_) treatment caused damage to sperm motility and viability (Chiou et al., 2008). The data shows that the sperm motility of the rats treated with the Sp alone (>86 to >91%) was found higher in the sampling days with increasing trend from Day 30 to Day 90 (Table 2). This result indicates that Sp feeding improved the sperm motility in rats compared to the arsenic induction group with increasing trend. Similarly, the sperm motility in all of the co-supplemented (iAs+Sp) groups (T3, T4 and T5) were found to be increased gradually from Day 30 up to Day 90. Therefore, the treatment of the arsenic induced rats with Sp improved (average 11.80%) the sperm motility and the highest dose of Sp (T5: 16.66% increased) was best among the doses of Sp in increasing sperm motility in the iAs-induced rats compared to the sole iAs treated rats (T1).

In this study, sperm morphology analysis revealed that the numbers (percent values) of morphological abnormalities of sperm (sperm with detached tail, coiled tail and sperm with bent tail) in iAs-induced rats (T1) increased gradually from Day 0 to Day 90. This finding suggests that iAs induction significantly increased the numbers of morphologically abnormal sperm in the trial rats with an increasing trend. Remarkably, it was observed that the numbers of the sperm with detached tail and that with bent tail in the control group were increased with time, except the sperm with coiled tail. The rats treated with the Sp (with or without iAs) showed significantly lower numbers (percent values) of all types of abnormal sperm compared to the control (T0) and iAs-induced (T1) groups. However, the rats of all the trial groups showed sperm with detached tail ranging from 0.00 to 3.38%; coiled tail: 0.00 to 2.78%; and with bent tail from 1.24 to 7.14%, which might also be at the acceptable levels. The findings reveal that iAs induction did not drastically affect the sperm morphology, and simultaneously, the Sp supplementation reduced the numbers of abnormal sperm on the sampling days (Chiou et al., 2008; Afkhami-Ardakani et al., 2018). The findings on reproductive parameters of the present study indicate that the iAs induction did not severely affect the sperm motility and morphology, but the decreased testicular weights were the significant adverse effect of the iAs induction in the trial rats. However, the majority cases of the Sp treatments recovered the testicular weights of the iAs-induced trial rats at the normal levels at least at the end of trial.

The dietary supplementation of Sp in iAs-induced rats positively improved sperm morphology, as manifested by an increase in sperm motility. This improvement may be due to the richness of Sp in zinc, which improves the activity of alkaline phosphatase enzyme in sperm (Barkallah et al., 2020). It was reported that *S. platensis* at a dose of 300 mg/kg was found to attenuate As-induced oxidative stress, testicular damage, and sperm abnormalities (Bashandy et al., 2016). In another study, Sp co-treatment with a dose of 300 mg/ kg bwt mitigated the arsenic induced toxicity in different organs of male rats (Ahmed et al., 2019). Oral administration of *S. platensis* at a dose rate of 500 mg/kg bw improved hepatic and renal antioxidant prospect (Abdel-Daim, 2014), and anti-inflammatory and anti-apoptotic potentials (Sadek et al., 2017) in rats. Furthermore, administration of *S. platensis* (@ 300 mg/kg bw) for four weeks could alleviate sperm quality and sex hormone alterations in furan treated rats (Abd El-Hakim et al., 2018). The dosing of Sp as dietary supplement has been advocated as safe food for human and animals use by several investigators, however, scant information is available on the mechanism of action of Sp in the treatment and management of chronic arsenicosis. Although, the mechanism by which iAs induces such drastic testes alterations is partially known and understood. Results from previous studies indicate that iAs exposure leads to the overproduction of ROS, oxidative DNA damage, apoptosis of testicular cells, and change in intra-testicular testosterone production (Barkallah et al., 2020; Bashandy et al., 2016). The protective effect of Sp supplementation in iAs-induced testicular degeneration may be attributed to the antioxidant effect of Sp under conditions of oxidative stress.

The present investigation showed that the treatment of the rats with Sp also affect the total As (tAs) contents in the testes of rats. The animals treated with iAs show increased amount of total arsenic (tAs) contents in the rat testes, on the contrary, dietary Sp supplementation significantly reduced the tAs load in the testes of the trial rats. It was evident that almost all of the doses of the Sp (1.0, 1.5 and 2.0 g/kg feed) reduced the tAs contents of the testicle of the iAs-induced rats. However, the highest (2.0 g/kg) dose of the Sp showed best efficacy in reducing (49.25%) the tAs contents from testicle of the iAs-induced trial rats. The supplementation of potential antioxidants like Sp to iAs induced animals seem to be beneficial for remedy of arsnicosis (Heck et al., 2007; Bashandy et al., 2016).

Moreover, it was found that compared to control rats, iAs treatment induce remarkable histopathological changes in the testicles including increased luminal areas and thinner germinal layers with markedly decreased spermatogenic population. Previous studies reported that iAs treatment also induce marked histological alterations in testis including reduction in the numbers of germ cell, vacuolization of seminiferous epithelium, spermatogenesis arrest, degeneration of Leydig cells and generation of ROS by reducing germ cell line viability (Barkallah et al., 2020; Yuan et al., 2014; Lima et al., 2018). Administration of Sp (1.0, 1.5 and 2 g/kg feed) significantly attenuated the severity of iAs-induced histological changes and effects on spermatogenesis, which can be attributed to its antioxidant activities. The co-supplementation of Sp+iAs showed nearly improved parameters compared to iAs group, but this improvement was stronger in sole Sp treated rats. These findings indicate that Sp antioxidant activities due to having several antioxidant compounds such as various vitamins, phycocyanin, selenium, polyunsaturated fatty acids are much more potent as also reported earlier in several studies (Yuan et al., 2014; Bashandy et al., 2016; Afkhami-Ardakani et al., 2018; Barkallah et al., 2020). Abd El-Baky et al. have demonstrated that Sp is able to increase its antioxidant activity during oxidative stress elevation and has a self-regulating antioxidant activity against the intensity of oxidative stress (Abd El-Baky et al., 2009). Furthermore, the dose of Sp supplementation also revealed significant role in reducing the numbers of seminiferous tubules with increased luminal areas and thinner germinal layers in the testicle of the iAs induced rats. The highest doses of (2.0 g/kg feed) was found as the most effective dose of Sp supplementation in protecting germ cell reduction, seminiferous epithelia vacuolization and spermatogenesis arrest. These findings corroborate with many of previous studies who reported the self-regulating antioxidant activity of Sp is more likely the reason of nonsignificant difference between low and high doses of Sp (Barkallah et al., 2020; Afkhami-Ardakani et al., 2018; Yuan et al., 2014). However, further study is necessary to reveal how spirulina mitigate the reproductive toxicity of arsenic in male rats, and enhance male fertility through biochemical and hormonal analysis.

## Conclusion

**Figure 4:**
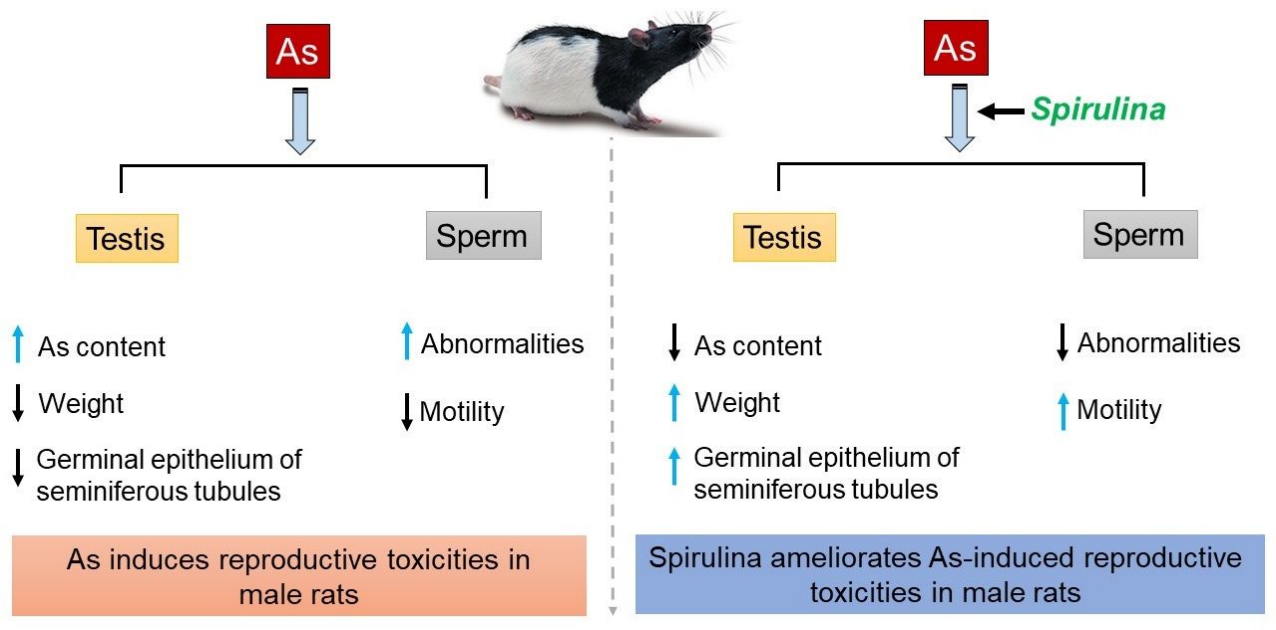
Summary of findings. Left panel: arsenic (As) induces reproductive toxicities in male rats by increasing total As contents and decreasing testicular weights and number of germinal epithelial layers of seminiferous tubules. As also increases number of abnormal sperm and reduces sperm motility. Right panel: Spirulina mitigates As-induced reproductive toxicities in male rats. Blue arrows (↑) indicate increased while black arrows (↓) indicate decreased.

The results of the present study suggest that dietary spirulina (*S*. *platensis)* supplementation improves arsenic induced reproductive toxicities by decreasing the total arsenic contents in testicles, increasing testicular weights and sperm motility, as well as number of morphologically normal sperm (Figure 4). Importantly, spirulina @ 2.0 g/kg feed was found as a potential remedy for reduction of adverse effects in testicular tissue and spermatogenic cells. Therefore, spirulina represents an effective agent to ameliorate the deleterious effects of arsenic induced reproductive toxicities in rats. Nonetheless, a further study is recommended to investigate the molecular mechanisms by which spirulina ameliorates the adverse effect of arsenic-induced reproductive toxicities in male.

## Supplementary Material

Supplementary material accompanies this paper.

Table S1 Total As in testicle of trial rats on sampling days (Day 0, 30, 60 and 90). Number of animals, n= 12/group.

This material is available as part of the online article from https://www.scielo.br/j/ar.
