# Supplementary material for: Spirulina ameliorates arsenic induced reproductive toxicity in male rats
Source: Anim Reprod. 2021 Nov 19;18(3):e20210035. doi: 10.1590/1984-3143-AR2021-0035 (PMC8628875; doi:10.1590/1984-3143-AR2021-0035)
Supplement: Table S1 [file 1806-9614-ar-18-3-e20210035-suppl01.pdf]

Table S1**:** Total As in testicle of trial rats on sampling days (Day 0, 30, 60 and 90). Number of animals, n= 12/group.

Groups of Animal	Total arsenic content in the testicle (mg/kg)			
	Day 0	*Day 30*	*Day 60*	*Day 90*
Control group (T0)	0.084±0.003	0.050±0.005^{b}	0.041±0.006^{d}	0.050±0.006^{d}
As-group (T1)<br>(% compared to T0 values)	0.075±0.006	2.644±0.103^{a} (5188.00)	2.895±0.265^{a} (6960.98)	2.989±0.265^{a} (5878.00)
Sp-group (T2)<br>(% compared to T0 values)	0.061±0.013	0.077±0.011^{b} (54.00)	0.181±0.004^{d} (341.46)	0.187±0.010^{d} (274.00)
As plus Sp group (T3)<br>(% compared to T1 values)	0.062±0.008	2.598±0.300^{a} (- 1.74)	1.886±0.284^{b} (- 34.85)	2.194±0.432^{b} (- 26.60)
As plus Sp group (T4)<br>(% compared to T1 values)	0.055±0.005	2.154±0.143^{ab} (- 18.53)	1.501±0.100^{c} (- 48.15)	1.662±0.325^{c} (- 44.40)
As plus Sp group (T5)<br>(% compared to T1 values)	0.048±0.002	1.618±0.048^{ab} (- 38.80)	1.463±0.245^{c} (- 49.46)	1.517±0.188^{c} (- 49.25)
LSD	ND	0.356	0.233	0.229
Level of Significance	ND	**	**	**

Data were presented as mean±SD; Values within parenthesis indicate percentage value; Values within parenthesis without any sign indicates increased percentage value; Values within parenthesis with '—' sign indicates decreased percentage value; ND = Analysis not done; ** = Significant at 1% level of probability. In a column values with similar superscript or without superscript do not differ significantly whereas values with dissimilar superscript differ significantly (as per DMRT).
